# Depressive symptoms improve over 2 years of type 2 diabetes treatment via a digital continuous remote care intervention focused on carbohydrate restriction

**DOI:** 10.1007/s10865-021-00272-4

**Published:** 2022-01-27

**Authors:** Rebecca N. Adams, Shaminie J. Athinarayanan, Amy L. McKenzie, Sarah J. Hallberg, James P. McCarter, Stephen D. Phinney, Jeffrey S. Gonzalez

**Affiliations:** 1 Virta Health Corp, 501 Folsom Street, San Francisco, CA 94105 USA; 2 Indiana University Health Arnett, Lafayette, IN USA; 3 Abbott Diabetes Care, Alameda, CA USA; 4 Department of Genetics, Washington University School of Medicine, St. Louis, MO USA; 5 Ferkauf Graduate School of Psychology, Yeshiva University, Bronx, NY USA; 6 Departments of Medicine (Endocrinology) and Epidemiology & Population Health, Albert Einstein College of Medicine, Bronx, NY USA

**Keywords:** Type 2 diabetes, Depression, Nutritional ketosis, Nutrition, Lifestyle intervention

## Abstract

Depressive symptoms are prevalent among people with type 2 diabetes (T2D) and, even at low severity levels, are associated with worse diabetes outcomes. Carbohydrate restriction is an effective treatment for T2D but its long-term impacts on depressive symptoms are unclear. In the current study we explored changes in depressive symptoms over 2 years among 262 primarily non-depressed T2D patients participating in a continuous remote care intervention emphasizing carbohydrate restriction. Subclinical depressive symptoms decreased over the first 10 weeks and reductions were maintained out to 2 years. Increased frequency of blood ketone levels indicative of adherence to low carbohydrate eating predicted decreases in depressive symptoms. Concerns have been raised with recommending restrictive diets due to potential negative impacts on quality-of-life factors such as mood; however, results of the current study support positive rather than negative long-term impacts of closely monitored carbohydrate restriction on depressive symptoms.

## Introduction

In the United States, over 30 million people have diabetes, which is recognized among the leading causes of morbidity and mortality (Ng et al., 2014; WHO, 2016). Treatment of type 2 diabetes (T2D) usually involves recommendations for significant lifestyle changes, and when these prove insufficient, medications are prescribed to manage the disease and slow progression. However, many patients fail to maintain glycemic targets after initiation of diabetes medication; in which case dosage increases and additional medications are often required (Turner et al., 1999). Medications contribute to patient burden in terms of cost, side effects such as weight gain, and increased risk of potentially life-threatening hypoglycemia (Gerstein et al., 2008; Hayes et al., 2006; Henry et al., 1993). Further, patients often have unfavorable views of polypharmacy and treatment intensification, especially with regards to initiation of insulin therapy (Hayes et al., 2006; Polonsky et al., 2011).

The burdens of diabetes treatment are also associated with emotional distress among individuals with T2D (Fisher et al., 2008; Perrin et al., 2017). Depression and elevations in subclinical depressive symptoms are more common among individuals with T2D compared to those without T2D (Fisher et al., 2008). Treatment with insulin therapy is associated with increased risk for depressive symptoms in T2D (Bai et al., 2018), suggesting that intensive treatment contributes to emotional distress. Furthermore, depressive symptoms are associated with worse self-management (e.g., missed medical appointments, non-adherence to medications and blood glucose monitoring) (Gonzalez et al., 2008; Hoogendoorn et al., 2019) and health outcomes (e.g., hyperglycemia, diabetes-related complications, and mortality) (de Groot et al., 2001; Lustman et al., 2000; Park et al., 2013). These impacts are not restricted to people with a depression diagnosis. Even among people with relatively low levels of depressive symptoms, the number and/or severity of these symptoms are incrementally associated with poorer diabetes self-management (Black et al., 2003; Gonzalez et al., 2007). Based on this evidence, current guidelines for the psychosocial care of individuals with diabetes emphasize the importance of identifying and addressing depression and diabetes-related emotional distress as a standard of comprehensive diabetes care (Young-Hyman et al., 2016).

There has been much research dedicated to understanding relationships between depressive symptoms and diabetes (Holt et al., 2014; Moulton et al., 2015; Tabák et al., 2014). The nature and directionality of the depression–diabetes relationship is hotly debated, but evidence points to a bi-directional relationship with both behavioral and physiological mechanisms. For instance, pathophysiological alterations (e.g., hypothalamic pituitary adrenal axis dysregulation, disturbance of circadian rhythms, pro-inflammatory processes) related to insulin resistance are common to both depression and T2D (Moulton et al., 2015). Additionally, high rates of depression and emotional distress could be attributed to the stress of living with diabetes (e.g., fear of progression and complications, day-to-day burden of self-management) (Gask et al., 2011; Stuckey et al., 2014).

## Carbohydrate restriction as treatment for T2D

Sustained dietary carbohydrate restriction is recognized as an alternative to increased pharmacological treatment of T2D in the standards of medical care (American Diabetes Association, 2021). Low-carbohydrate diets have consistently elicited improvements in HbA1c, weight, inflammation and cardiovascular risk factors while reducing reliance on antiglycemic medications (Athinarayanan et al., 2019; Forsythe et al., 2007; Saslow, Daubenmier et al., 2017; Saslow, Mason et al., 2017; Saslow et al., 2018; Westman et al., 2008; Yamada et al., 2014). Despite evidence for improved diabetes outcomes up to 1 and 2 years, questions have been raised about the sustainability of the approach based on concerns relating to the burdens of adhering to the restrictive diet and the potential negative impact on patient well-being. However, the impact of carbohydrate restriction on well-being in T2D is understudied.

## Carbohydrate restriction and mood

A burgeoning literature does not support any negative impacts of carbohydrate restriction on mood. In fact, there is a small but growing literature focused on potential mood stabilizing properties of the diet, particularly among individuals with bipolar disorder (Brietzke et al., 2018; Campbell & Campbell, 2019; El-Mallakh & Paskitti, 2001; Norwitz et al., 2020). Researchers theorize that changes in the brain (e.g., the switch to utilizing ketones for energy instead of glucose) could affect mood. In addition, a handful of studies have explored the impact of a low-carbohydrate diet on mood and other psychological outcomes among patients without mental health conditions or diabetes. The majority of studies identified either modest improvements or no change in mood or other indicators of psychological well-being (Breymeyer et al., 2016; Brinkworth et al., 2009; D’Anci et al., 2009; Halyburton et al., 2007; Harvey et al., 2019; McClernon et al., 2007; Rosen et al., 1985). A few longitudinal studies identified short-term improvement followed by regression to baseline mood (Brinkworth et al., 2009; Halyburton et al., 2007; Rosen et al., 1985).

However, there are many reasons to expect that a low-carbohydrate diet could have a positive effect on mood in people with T2D and other metabolic conditions. First, higher glucose and inflammation are linked to metabolic syndrome, diabetes, and depression (Moulton et al., 2015; Stuart & Baune, 2012); thus, improvements in glucose and inflammation observed with a low-carbohydrate diet could have a positive influence on depressive symptoms. For example, data from the Diabetes Prevention Program suggest that it is not the diagnosis of diabetes that increases risk for depression, but rather increases in HbA1c and fasting blood glucose that follow diagnosis (Marrero et al., 2015). Furthermore, improvements in HbA1c have been associated with decreased depressive symptoms in adults with T2D (de Groot et al., 2012). Second, the majority of people with T2D have obesity (Daousi et al., 2006) and the low-carbohydrate diet is associated with clinically significant long-term weight loss (Gardner et al., 2007; Sackner-Bernstein et al., 2015; Shai et al., 2008). Substantial weight loss such as that experienced following bariatric surgery is associated with improvement in depressive symptoms (Strain et al., 2014; Zwaan et al., 2011). Third, a low-carbohydrate diet tends to reduce dependence on antiglycemic medications, which is aligned with patient preferences and avoids negative secondary consequences of intensive therapy regimens, including risk of severe hypoglycemia and reduced well-being.

A few small studies have examined changes in psychological well-being among people with prediabetes or T2D in the context of a low-carbohydrate diet. Results were mixed, with some indicators of psychological well-being (e.g., diabetes distress) improving and others staying the same (e.g., depressive symptoms) (Guldbrand et al., 2014; Saslow et al., 2014; Saslow, Daubenmier et al., 2017; Saslow, Mason et al., 2017). However, the sample sizes in these small studies and time-limited carbohydrate restriction may have reduced power for these analyses of secondary outcomes.

## Current study

Given the many reasons people with T2D may experience mood benefits from a low-carbohydrate diet, we conducted a secondary analysis of 2-year data from an ongoing 5-year clinical trial of a continuous remote care intervention (CCI) to explore changes in depressive symptoms. As part of the CCI, participants were coached in carbohydrate restriction with the goal of achieving nutritional ketosis (i.e., ketones, assessed via blood beta-hydroxybutyrate (BHB), ≥ 0.5 mM) to improve metabolic outcomes. The 10-week, 1-year, and 2-year primary outcomes were previously published (Athinarayanan et al., 2019; Hallberg et al., 2018; McKenzie et al., 2017). At 2 years, HbA1c decreased by 0.9% from 7.7% concurrent with significant reductions in the number and dosage of diabetes medications prescribed. Weight was also reduced by 12% at 1 year and 10% at 2 years. Inflammatory markers and most cardiovascular disease risk factors improved. In addition to metabolic outcomes, we assessed participants’ depressive symptoms at baseline, 10 weeks, 1 year, and 2 years.

Three aims guided the current analyses. First, we assessed changes in depressive symptoms over time via the average change in the total symptom severity score and the change in the percentage of participants meeting clinical cut-offs for depression. Second, we aimed to identify potential predictors of change in depressive symptoms (i.e., BHB values; change in weight, HbA1c, medication use, and inflammation) to better understand why they might be occurring. Third, we explored whether participants with evidence of baseline or historical depressive symptoms experienced the same metabolic benefits (i.e., improvements in HbA1c, weight, HOMA-IR, and fasting glucose) as participants who did not, given the links between depression and treatment adherence/engagement (Marcus et al., 1992).

## Methods

### Participants and procedure

The detailed study design and primary outcomes were previously published (Athinarayanan et al., 2019; Hallberg et al., 2018; McKenzie et al., 2017), and the current results are based on a 2-year post hoc analysis using the data collected from the same cohort (Clinicaltrials.gov identifier: NCT02519309). Briefly, this is a non-randomized, open-label study including patients 21–65 years of age with a diagnosis of T2D and a body mass index of > 25 kg/m^2^. Participants self-selected to receive the continuous care intervention (CCI) or usual care. All study participants provided written informed consent and the study was approved by the Franciscan Health Lafayette Institutional Review Board, Lafayette, IN, USA. No changes in metabolic parameters were observed in the usual care cohort over 2 years, so we limited this analysis to further our understanding of the CCI.

The 262 CCI participants were provided access to a web-based software application (app) and initially advised to achieve nutritional ketosis (blood BHB level of 0.5–3.0 mmol L^−1^) through sufficient carbohydrate restriction (initially < 30 g/day but gradually increased based on personal carbohydrate tolerance and health goals). Participants used the app to communicate with their remote care team consisting of a health coach and a medical provider. The remote care team provided education and support regarding dietary changes, behavior modification techniques for maintenance of lifestyle changes, and directed medication changes for diabetes and anti-hypertensive medications. The participants used the app to upload and monitor their biometric status including body weight, blood glucose, and blood BHB. Biomarkers allowed for daily feedback to the care team and individualization of patient instruction.

The majority of the intervention was continuous and remote, but participants completed in-person study visits involving physical exam, laboratory, and body composition measures at baseline, 10 weeks following dietary change, and 1 and 2 years following enrollment.

### Measures

#### Baseline demographic and medical characteristics

Age, race, sex, and time since diabetes diagnosis were obtained from electronic medical records.

#### Medication use

All medication use and dosage, including antihyperglycemic and antidepressant medications, were tracked continuously by the participants’ assigned medical provider in the app. We extracted participants’ medication use data at the specified time points. For the number of diabetes-specific medications, we summed the number of medication classes from which they were prescribed medication. Diabetes-specific medication classes included sulfonylureas, insulin (long-acting, short-acting, intermediate-acting, and human separately), thiazolidinediones, SGLT-2 inhibitors, DPP-4 inhibitors, and GLP-1 agonists.

#### Depressive symptoms and diagnoses

Depressive symptoms were measured during clinic visits using the 20-item Center for Epidemiological Studies Depression Scale (CES-D; Radloff, 1977). For continuous assessment of depressive symptoms, we summed 14 items for a total score based on prior research which supports the removal of 6 items (Carleton et al., 2013; Carter et al., 2016). The 14-item version of the CES-D has excellent reliability and validity evidence in the general population and among people with T2D. A sample item is “I felt that I could not shake off the blues even with help from my family and friends.” Responses range from 0 (Rarely or none of the time) to 3 (Most or all of the time). In the current sample, internal consistency for the 14 items was excellent (α = 0.86) at baseline. We also looked at clinical cut-offs for depression using the full 20-item CES-D since cut-offs were established using that version; we used a clinical cut-off of 22 based on research showing that cut-off is most similar to established cut-offs on the PHQ-9 and Beck Depression Inventory (Choi et al., 2014) and a better indicator of Major Depressive Disorder in people with T2D (Fisher et al., 2007) than the original CES-D cut-off of 16. Additionally, we conducted a retrospective review of participants’ external medical charts to identify any depressive disorder diagnoses documented before their study enrollment. Among participants with a depressive disorder diagnosis in their medical chart at baseline, only 33% met the CES-D clinical cut-off at baseline. Among those who met the clinical cut-off at baseline, only 42% had a diagnosis in their medical chart. Among those *not* meeting the clinical cut-off at baseline, 15% still had a diagnosis in their medical chart.

#### Metabolic and inflammatory markers

Blood analytes (fasting glucose, insulin, HbA1c, c-reactive protein, and white blood cells) were determined via standard procedures at a Clinical Laboratory Improvement Amendment (CLIA) accredited laboratory on the day of sample collection or from stored serum. Insulin-derived HOMA-IR (homeostatic model assessment- insulin resistance) was calculated (Matthews et al., 1985). Weight was measured in the clinic.

#### Blood ketones

Beta-hydroxybutryate (BHB) was measured via lab testing from blood samples taken during clinic visits. Additionally, participants were asked to measure their ketones regularly via blood fingerstick (Abbott Precision Xtra) and enter the values into the mobile app. Ketones were generally entered daily during the first 10 weeks. The percentage of participant-entered ketone values reaching 0.5 mM or higher (indicative of nutritional ketosis) was computed to estimate adherence to carbohydrate restriction.

### Statistical analyses

First, we examined the assumptions of normality and linearity. According to Kline’s (2011) guidelines, 2 variables (i.e., HOMA-IR and c-reactive protein) were positively skewed. To handle the skewed variable, we removed extreme outliers (i.e., the top 1% of values). We chose this approach rather than a transformation because interpretation is more difficult with transformed variables, and results did not differ based on the approaches (Athinarayanan et al., 2019).

Next, descriptive statistics were computed to characterize the sample. In addition, independent *t*-tests were conducted to compare 2-year completers and participants who dropped out by 2 years on baseline values of all study variables. No differences were found (see 2-year outcomes paper for most results; Athinarayanan et al., 2019), except participants who dropped out by 2 years reported greater depressive symptoms at baseline. Thus, to handle missing data we used multiple imputation and included baseline depressive symptoms as one of the predictors in the imputation model (Moons et al., 2006). Missing values were estimated from 40 imputations (Graham et al., 2007) from multiple regression. Multiple imputation provides reasonably unbiased estimates if data are missing at random (Baraldi & Enders, 2010).

To assess changes in depressive symptoms over time (Aim 1), we used two approaches. First, we performed a linear mixed-effects model (LMM) using SPSS statistical software which included a fixed effect for time. The outcome variable was the total score of the 14-item version of the CES-D. Covariates for this model and all LMMs in the study included baseline age, sex, race (African American vs. other), years since diabetes diagnosis, insulin use, and antidepressant use. An unstructured covariance structure was specified for all LMMs to account for correlations between repeated measures. Second, we examined changes in the percentage of participants meeting the clinical cut-off for depression (score of 22 or higher on the 20-item version of the CES-D) from baseline to each follow-up time point using McNemar’s tests.

The goal of Aim 2 was to explore potential predictors of change in depressive symptoms over time. First, we conducted paired *t*-tests to confirm that the hypothesized predictors (i.e., lab BHB, weight, HbA1c, insulin dose, medication use, c-reactive protein, and white blood cells) changed over time. Second, we conducted General Linear Model (GLM) analyses to assess whether each variable that changed over time was associated with change in depressive symptoms. Specifically, we conducted six separate GLM analyses to assess whether improvement in each of the six predictor variables that changed over the first 10 weeks was associated with follow-up depressive symptoms, controlling for baseline depressive symptoms and the same covariates included in the LMM analysis in Aim 1. "Improvement" in each predictor was defined as follows: percent change in weight from baseline to 10 weeks; percent change in HbA1c from baseline to 10 weeks; percentage of BHB values ≥ 0.5 mM entered into the app over the first 10 weeks; percent change in insulin dose from baseline to 10 weeks among those taking it at baseline; percent change in the number of diabetes-specific medication classes prescribed from baseline to 10 weeks; and fold change in inflammatory markers from baseline to 10 weeks.

For Aim 3, we explored whether historical or baseline depression status predicted diabetes outcomes at 2 years. We used three proxies for depression status: (1) meeting the clinical cut-off for depression on the CES-D at baseline; (2) any historical depressive disorder diagnosis noted in the external medical chart; and (3) prescription of an antidepressant medication at baseline. Similar to Aim 1, for Aim 3 we conducted LMMs. Outcomes included HbA1c, insulin-derived HOMA-IR, weight, and fasting glucose. Twelve separate LMMs included fixed effects for time, depression status (e.g., meets clinical cut-off of 22 versus not), and a time by depression status interaction.

## Results

### Participant characteristics

Table 1 presents baseline characteristics of the 262 CCI participants. Participants’ average depressive symptom severity score was 9.4, indicating little to no depressive symptoms on average. This average score is slightly lower than a similar sample of T2D patients (11.1; Carter et al., 2016). About 15% met the clinical cut-off for depression at baseline, whereas 19.5% had any depressive disorder recorded in their medical chart. About 61% of participants who met the clinical cut-off at baseline were prescribed an antidepressant medication, compared to almost 38% in the full sample. Among those prescribed an antidepressant at baseline, only 25% met the clinical cut-off for depression. Antidepressant use was not systematically adjusted by study providers and did not change significantly over the study period.

**Table 1:** Participant characteristics

Baseline characteristic *(N* = *262)*	Baseline		
	*N* (%)	M (SD)	Range
Average age (years)		53.8 (8.4)	28.0–66.0
African American	18 (6.9)		
Body mass index		40.4 (8.8)	24.8–86.9
Female	175 (66.8)		
Years since type 2 diabetes diagnosis		8.4 (7.2)	0.0–33.0
Depressive disorder documented in external chart	51 (19.5)		
Meets clinical cut-off for depression	36 (15.1)		
Antidepressant prescribed	99 (37.8)		
Beta-hydroxybutyrate (mmol·L^−1^)		0.17 (0.15)	0.04–1.07
Insulin prescribed	78 (29.8)		
Insulin dosage (among those prescribed)		90.6 (69.2)	5.0–405.0
Number of diabetes-specific medications prescribed		1.7 (1.1)	0.0–5.0
White blood cells (k/cumm)		7.2 (1.9)	3.4–14.7
C-reactive protein (nmol L^−1^)		7.6 (6.71)	3.5–13.3

*M * mean, *SD* standard deviation

### Aim 1: Do depressive symptoms change over time?

**Fig. 1:**
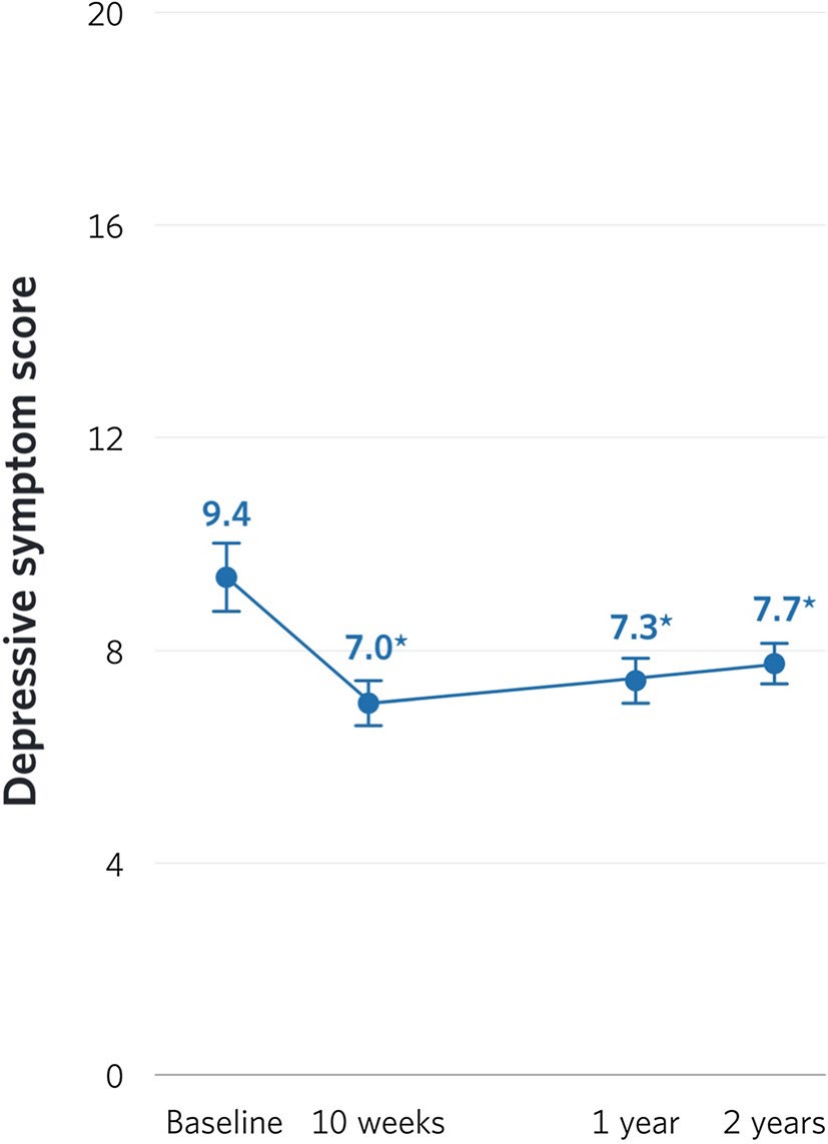
Change in depressive symptoms over 2 years. Asterisks indicate significant change from baseline (all *p*s < 0.0001)

In the LMM analysis, the overall effect of time was significant (*p* < 0.0001), such that depressive symptoms decreased over time (Fig. 1). Specifically, depressive symptoms decreased from baseline (M = 9.4, SE = 0.47) to 10 weeks (M = 7.0, SE = 0.40, *p* < 0.0001), 1 year (M = 7.3, SE = 0.49, *p* < 0.0001), and 2 years (M = 7.7, SE = 0.54, *p* < 0.0001). After the initial 10 weeks, no significant changes in depressive symptoms were observed (*p*s > 0.05).

Based on McNemar’s tests, the percentage of participants that met the clinical cut-off for depression decreased from baseline to 10 weeks ( −11.5% from 18.4%, *p* = 0.001) and baseline to 2 years ( −4.3% from 15.0%, *p* = 0.04). The decrease from baseline to 1 year was not statistically significant (*p* > 0.05). Baseline percentages differ from analysis to analysis because of multiple imputation.

### Aim 2: What factors are associated with change in depressive symptoms?

Given that depressive symptoms changed between baseline and 10 weeks and thereafter remained statistically unchanged, we explored predictors of change during that time period only. First, we confirmed that all our hypothesized predictors changed from baseline to 10 weeks. As expected, significant changes were observed in weight ( −19.1 lbs from 256.8 lbs, *p* < 0.0001), HbA1c ( −1.1% from 7.6%, *p* < 0.0001), lab BHB (+ 0.4 from 0.2 mM, *p* < 0.0001), insulin dose ( −58.7 from 91.2 units per day, *p* < 0.0001), number of diabetes-specific medications prescribed ( −0.5 from 1.7, *p* < 0.0001), and white blood cells (+ 0.50 from 7.3 k/cumm, *p* < 0.0001). The change in c-reactive protein was not yet significant at 10 weeks (*p* = 0.05).

Next, we conducted six separate GLM analyses to assess whether change in each predictor (except c-reactive protein) was associated with change in depressive symptoms from baseline to 10 weeks. The only significant predictor of change in depressive symptoms was percent of reported BHB values ≥ 0.5 mM, such that participants who logged a greater percent of BHB values at or above 0.5 mM had greater reductions in depressive symptoms from baseline to 10 weeks (*b* =  −2.4, *p* = 0.02).

### Aim 3: Does baseline depression status predict glycemic outcomes?

We conducted 12 LMMs to assess whether depression status predicted weight, HbA1c, HOMA-IR, and fasting glucose over 2 years. When depression status was defined by meeting the clinical cut-off at baseline, the main effect of baseline depression status was not significant for any metabolic outcomes (*p*s > 0.05). When depression status was defined by a depressive disorder diagnosis in the external medical chart, participants with depression had higher HbA1cs (*p* = 0.02), HOMA-IRs (*p* = 0.006), and fasting glucoses (*p* < 0.0001) on average over the 2 years. Although participants with a history of depression had worse glycemia and insulin resistance on average, the pattern of change mirrored that of participants without depression such that glycemia and insulin resistance improved over time (see Table 2 for means). The average weight did not differ between the groups over the 2 years (*p* > 0.05). When depression status was defined as being prescribed an antidepressant medication at baseline, the main effect of depression was significant for HOMA-IR (*p* = 0.04) but not any other metabolic outcomes (*p*s > 0.05). Participants prescribed an antidepressant at baseline had worse insulin resistance on average over the 2 years, although the pattern of change was similar between groups.

**Table 2:** Metabolic outcome means by depression status and time

	Baseline	10 Weeks	1 Year	2 Years
	Mean ± SE	Mean ± SE	Mean ± SE	Mean ± SE
HbA1c (%)				
Meets clinical cut-off	7.85 ± 0.24	6.46 ± 0.17	6.19 ± 0.16	6.87 ± 0.23
Doesn’t meet clinical cut-off	7.56 ± 0.10	6.51 ± 0.07	6.22 ± 0.07	6.62 ± 0.09
Diagnosis in chart	8.05 ± 0.20	6.72 ± 0.15	6.37 ± 0.16	6.84 ± 0.21
No diagnosis in chart	7.50 ± 0.10	6.45 ± 0.07	6.18 ± 0.07	6.61 ± 0.09
Prescribed antidepressant	7.61 ± 0.15	6.52 ± 0.11	6.21 ± 0.10	6.64 ± 0.14
Not prescribed antidepressant	7.60 ± 0.11	6.49 ± 0.08	6.21 ± 0.08	6.67 ± 0.11
HOMA-IR				
Meets clinical cut-off	9.43 ± 1.09	5.32 ± 0.86	5.01 ± 0.74	6.85 ± 0.90
Doesn’t meet clinical cut-off	8.82 ± 0.42	5.89 ± 0.35	4.85 ± 0.30	5.05 ± 0.37
Diagnosis in chart	10.33 ± 0.95	6.45 ± 0.78	5.74 ± 0.63	6.56 ± 0.79
No diagnosis in chart	8.56 ± 0.42	5.65 ± 0.36	4.66 ± 0.30	5.02 ± 0.37
Prescribed antidepressant	9.86 ± 0.64	5.77 ± 0.55	5.55 ± 0.47	5.76 ± 0.55
Not prescribed antidepressant	8.33 ± 0.49	5.83 ± 0.40	4.46 ± 0.35	5.06 ± 0.44
Fasting glucose (mg/dL)				
Meets clinical cut-off	175.94 ± 9.78	128.10 ± 5.83	125.21 ± 6.61	142.54 ± 9.02
Doesn’t meet clinical cut-off	158.23 ± 4.06	129.33 ± 2.34	123.97 ± 2.59	130.67 ± 3.75
Diagnosis in chart	186.31 ± 8.34	132.64 ± 5.18	131.19 ± 5.91	137.67 ± 8.57
No diagnosis in chart	154.73 ± 4.06	128.30 ± 2.37	122.46 ± 2.61	131.19 ± 3.72
Prescribed antidepressant	158.27 ± 6.03	129.16 ± 3.54	126.29 ± 4.18	133.62 ± 5.58
Not prescribed antidepressant	162.48 ± 4.75	129.14 ± 2.69	122.89 ± 2.95	131.76 ± 4.37
Weight (pounds)				
Meets clinical cut-off	263.07 ± 9.15	239.39 ± 9.06	231.89 ± 8.28	231.09 ± 9.45
Doesn’t meet clinical cut-off	255.58 ± 3.71	237.33 ± 3.62	224.69 ± 3.46	225.80 ± 3.55
Diagnosis in chart	258.54 ± 7.83	239.88 ± 7.68	230.33 ± 7.40	232.75 ± 7.54
No diagnosis in chart	256.26 ± 3.82	237.09 ± 3.71	224.66 ± 3.50	225.09 ± 3.64
Prescribed antidepressant	261.17 ± 5.62	240.84 ± 5.53	229.59 ± 5.27	231.49 ± 5.50
Not prescribed antidepressant	254.02 ± 4.37	235.71 ± 4.25	223.47 ± 4.02	223.64 ± 4.06

SE = standard error. HOMA-IR = homeostatic model assessment- insulin resistance. Means and standard errors reported for LMM outcomes are estimates obtained from linear mixed-effects models. These are intent-to-treat analyses that provide adjusted means, controlling for baseline age, sex, race, years since diabetes diagnosis, insulin use, and antidepressant medication use (except antidepressant medication use was not a covariate in the models comparing outcomes between participants with and without an antidepressant prescription). All metabolic outcomes significantly improved from baseline to 10 weeks, 1 year, and 2 years for all groups. Significant group differences by depression status included: (1) HbA1c at baseline between those with and without a depression diagnosis in the external chart; (2) HOMA-IR at baseline and 2 years between those with and without a depression diagnosis in the external chart; (3) HOMA-IR at 1 year between those prescribed and not prescribed an antidepressant at baseline; (4) fasting glucose at 10 weeks and 1 year between those with and without a depression diagnosis in the external chart

## Discussion

During the first 2 years of an intervention emphasizing carbohydrate restriction for glycemic control, T2D participants’ mood improved. Depressive symptoms were reduced from baseline to all follow-up time points over the 2 years examined. Improvements occurred over the first 10 weeks of the intervention and then stabilized statistically, although a slight regression was observed from 10 weeks to 2 years. This is consistent with other studies of low-carbohydrate and other dietary interventions in that mood did not worsen and improvements tended to occur early in treatment (Brinkworth et al., 2009; Halyburton et al., 2007; Harvey et al., 2019; McClernon et al., 2007; Rosen et al., 1985). However, depressive symptoms appeared to regress less in the current study than other dietary studies despite the longer follow-up period. The continuous care model and sustained metabolic improvements (see Table 2 for adjusted means over time) may have contributed to the overall stability of improvements over 2 years.

Although the average change in depressive symptoms was small in this sample of adults who typically did not present with clinically significant depressive symptoms, small changes in depressive symptoms in this population are clinically important as they have been associated with better health outcomes (Feltz-Cornelis et al., 2010). Furthermore, the decreased percentage of participants meeting clinical cut-offs for depression lends evidence of clinical significance. The percentage of participants meeting the cut-off decreased from baseline to 10 weeks and 2 years but not 1 year. Given the slight regression from 10 weeks to 1 and 2 years, non-significant result at 1 year, and higher *p*-value at 2 years (*p* = 0.04), the most conservative conclusion is that reductions in probable depression diagnoses were limited to 10 weeks, although improvements in the symptom severity score were sustained for 2 years.

Because the CCI was not designed to target depressive symptoms specifically, we explored potential predictors of symptom improvement over the first 10 weeks. The percentage of daily ketone values reaching the nutritional ketosis threshold (indicating dietary adherence) was the only significant predictor of improvement in depressive symptoms from baseline to 10 weeks. To our knowledge, this is the first analysis to leverage daily ketone measurements to estimate the percentage of time participants were in nutritional ketosis and its relation to changes in mood over an extended period of time. However, this relationship is consistent with current theories regarding the potential role of ketones in mood stabilization. This is also consistent with results of the Saslow, Daubenmier et al. (2017), Saslow, Mason et al. (2017)) study in which participants with T2D who were assigned to the low-carbohydrate diet reported “liking how they felt” better than participants in other dietary conditions in a study questionnaire.

Despite literature linking weight, HbA1c, medication use, and inflammation to depressive symptoms, changes in these factors were not associated with improvement in depressive symptoms in this study. One potential explanation for the non-significant results is that qualitative perceptions of health improvement and success may be the biggest drivers of improvement in depressive symptoms. If a qualitative perception of health improvement (or psychological experiences that relate to it such as sense of control and accomplishment regarding diabetes or decreased worry about long-term complications) drives improvements in depressive symptoms, then results may be non-significant because multiple factors impact that perception. For example, a participant who experiences improvements in at least one area (e.g., HbA1c) may perceive health improvement even if changes were not seen in other areas (e.g., weight). A different combination of changes may promote this perception of success in each participant, preventing detection of effects for individual predictors. However, despite this challenge, we may have been able to detect an effect for ketones because they are the most proximal and reliable indicator of dietary adherence and hypothesized to be a cause of the improvements in all the other predictor variables (Arase et al., 1988; Ari et al., 2019; Newman & Verdin, 2014; Youm et al., 2015). Additional research is needed to better understand how all of these factors are related to mood and other aspects of health-related quality of life over time.

In preliminary analyses we discovered that baseline depression status predicted study dropout, which is consistent with studies of other lifestyle interventions (Sanderson & Bittner, 2005; Stubbs et al., 2016). To manage this bias in our data, we included baseline depressive score as a predictor in the multiple imputation model, which has been found to produce estimates very close to “true” values in a simulation (Moons et al., 2006). However, this is a limitation that must be considered in interpreting the results for aim 3 especially. For aim 3 we were interested in whether baseline depression status would predict primary metabolic outcomes (i.e., weight, HbA1c, HOMA-IR, fasting glucose) over 2 years and results depended on the way depression was defined. Given the imperfect definitions of depression, it is unsurprising that results varied. While meeting the clinical cut-off at baseline did not predict any outcomes, having a historical chart diagnosis of depression predicted worse glycemia and insulin resistance on average over the 2 years. However, the pattern of change was similar among those with and without a history of depression in that glycemia and insulin resistance improved overall over the 2 years in both groups. Baseline antidepressant use only predicted worse insulin resistance on average, which also improved similarly in both groups over the 2 years. While it is promising that results suggest the CCI is still effective for participants presenting with depression, future efforts should focus on retaining more of these participants. Research is required to better understand why participants with depression drop out at higher rates, and whether changes could be made to the CCI to better accommodate them. For example, participants with depression may require more health coach support or additional skills-building (e.g., cognitive behavioral therapy skills which have improved both depression and adherence to T2D treatment in prior research) (Safren et al., 2014) to prevent drop out. Another potential strategy is to systematically identify people with depression and connect them to high-quality depression treatment.

Limitations of this study and directions for future research should be noted. First, this was a single-arm exploratory analysis; although we hypothesize that changes in depressive symptoms resulted from the CCI, our study design does not enable us to draw any causal conclusions. Second, the CCI is multi-component making it hard to disentangle potential impacts of the dietary versus support components. Third, the sample lacked racial diversity limiting generalization of the results to all T2D patients. The sample was also relatively non-depressed and although this increased generalizability of the findings, it made it harder to detect potential effects on depression as non-depressed participants have less opportunity for improvement. Among the small subset of participants (15%) who met the clinical cut-off for depression at baseline, depressive symptoms appeared to decrease more dramatically over the 2 years (about 6 points in the depressed subset compared to about 2 points in the full sample). Future research should explore changes in depressive symptoms in a more racially diverse sample and among different subsets of the current sample, such as those with confirmed depression diagnoses. Finally, although depressive symptoms as measured by the CES-D are very important to assess, there are other diabetes-specific health-related quality of life factors that would be valuable to assess in this context. For example, diabetes distress is thought to be even more prevalent in this population than depressive symptoms and may be more strongly associated with diabetes management behaviors and outcomes (Fisher et al., 2010). Diabetes distress and other diabetes-specific quality of life factors such as fear of hypoglycemia and fear of diabetes complications may be impacted by the CCI and should receive attention in future research.

### Summary

Depressive symptoms are common and associated with worse outcomes in people with T2D, making mood an important consideration for T2D treatment. Evidence suggests that intensive medication treatment for T2D may have a negative effect on patient mood and well-being (Huang et al., 2007). Furthermore, intensive treatments are often less preferred by patients who commonly value reduced need for medication as an outcome of successful diabetes management. Carbohydrate restriction offers a promising approach for improved diabetes outcomes, based on previously reported findings. The current analysis reveals that participants’ depressive symptoms consistently improved over 2 years of a digital T2D treatment emphasizing a carbohydrate-restricted eating plan. Additionally, greater adherence to low carbohydrate eating predicted decreased depressive symptoms. Unlike other lifestyle intervention studies, significant improvements were sustained long-term up to 2 years. This comprehensive continuous remote care model, which facilitated sustained dietary adherence and metabolic benefits, may have enabled long-term improvements in depressive symptoms among people with T2D participating in the intervention.

## Data Availability

Data is not available to share due to participant privacy concerns.
